# The Bubble Box: Towards an Automated Visual Sensor for 3D Analysis and Characterization of Marine Gas Release Sites

**DOI:** 10.3390/s151229825

**Published:** 2015-12-05

**Authors:** Anne Jordt, Claudius Zelenka, Jens Schneider von Deimling, Reinhard Koch, Kevin Köser

**Affiliations:** 1 GEOMAR Helmholtz Centre for Ocean Research Kiel, Kiel 24148, Germany; jschneider@geomar.de (J.S.V.D.); kkoeser@geomar.de (K.K.); 2 Department of Computer Science, Kiel University, Kiel 24118, Germany; cze@informatik.uni-kiel.de (C.Z.); rk@mip.informatik.uni-kiel.de (R.K.)

**Keywords:** bubbles, methane, 3D reconstruction, stereo, underwater photogrammetry, flux, size distribution, rise speed

## Abstract

Several acoustic and optical techniques have been used for characterizing natural and anthropogenic gas leaks (carbon dioxide, methane) from the ocean floor. Here, single-camera based methods for bubble stream observation have become an important tool, as they help estimating flux and bubble sizes under certain assumptions. However, they record only a projection of a bubble into the camera and therefore cannot capture the full 3D shape, which is particularly important for larger, non-spherical bubbles. The unknown distance of the bubble to the camera (making it appear larger or smaller than expected) as well as refraction at the camera interface introduce extra uncertainties. In this article, we introduce our wide baseline stereo-camera deep-sea sensor bubble box that overcomes these limitations, as it observes bubbles from two orthogonal directions using calibrated cameras. Besides the setup and the hardware of the system, we discuss appropriate calibration and the different automated processing steps deblurring, detection, tracking, and 3D fitting that are crucial to arrive at a 3D ellipsoidal shape and rise speed of each bubble. The obtained values for single bubbles can be aggregated into statistical bubble size distributions or fluxes for extrapolation based on diffusion and dissolution models and large scale acoustic surveys. We demonstrate and evaluate the wide baseline stereo measurement model using a controlled test setup with ground truth information.

## 1. Introduction

**Figure 1:**
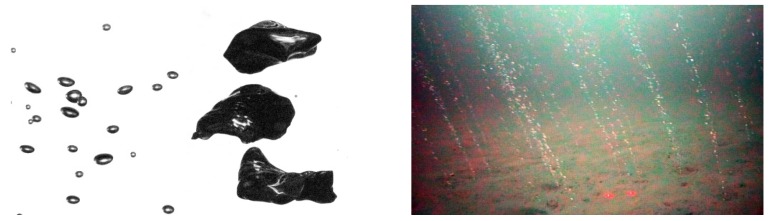
The shape and speed of gas bubbles rising in a liquid depends on the bubbles’ volume and surface area and other (physical and chemical) properties of gas and liquid [3]. For instance, air bubbles in water are almost spherical up to millimeter size (small bubbles to the **left**), become approximately ellipsoidal in the several millimeter range and can be irregularly shaped when much larger. The image to the **right** shows an area in the North Sea where methane is released from the ocean floor and forms distinct bubble streams. Measuring the flux, the rise speed, and the bubble size distribution is of crucial importance for larger scale ocean and atmosphere models.

The oceans’ seafloors host significant amounts of the greenhouse gas methane in the form of solid gas hydrate, dissolved in sedimentary pore water, or as free gas. The occurrence of free methane gas in the seabed and respective release into the water column in the form of rising gas bubbles represents a global phenomenon termed *gas seepage* (cf. to [1,2], see also Figure 1).

**Figure 2:**
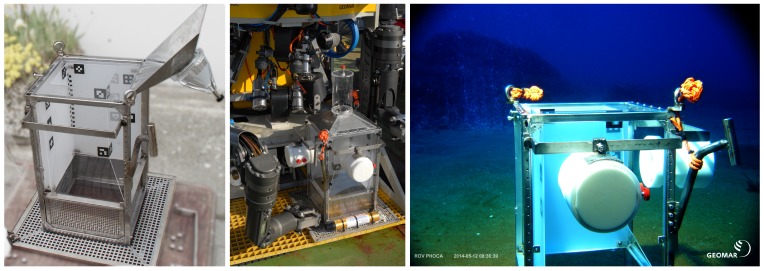
Three views of the bubble box, from **left** to **right**: box without cameras during development, box on ROV Phoca before being deployed (research cruise on RV Poseidon) and box in shallow water near Panarea, an area with heavy carbon dioxide release.

Methane, however, represents a strong greenhouse gas on Earth. Consequently, if methane release from the seabed migrates towards the atmosphere, it then represents a threat in terms of global warming [2,4], but even very rough estimates for the oceans’ contributions to the atmosphere’s methane budget are very challenging. The majority of known marine gas seepages originates from microbial degradation of organic matter into methane. Subsequently, the major fraction of methane is filtered *in situ* by a complex microbial community on the seabed [5]. However, gas bubbles bypass this benthic filter enabling a highly efficient transport of CH${}_{4}$ from the sediment into the water column and ultimately to the atmosphere. To a lesser degree, the release of gas from the seafloor is associated with sub-seabed volcanic activity and emission of abiotic CO${}_{2}$, methane, and H${}_{2}$S gas [6]. Recent work also discusses anthropogenic contributions of gas leakage from the seafloor by marine oil or gas exploitation activities, carbon storage and enhanced oil recovery facilities, gas pipelines, and abandoned wells [7,8]. After gas bubbles release from the seafloor into the water column, the bubbles undergo diffusion with ambient seawater. During their rise with decreasing hydrostatic pressure, gas bubbles may lose large amounts of their initial amounts of moles, e.g., driven by undersaturation of seawater with methane. Numerical models are available to predict the diffusion and dissolution of gas bubbles in seawater and allow for calculation of bubble lifetime, mole fractions, and rise height of seepage gas bubbles [9,10]. The input parameters initial gas bubble size, water depth, gas mole fraction, and environmental properties of the ambient seawater are required for modeling the chemical bubble evolution and lifetime. However, only a very limited number of studies investigated the crucial parameter of bubble size distribution so far [11,12]. So called gas hydrate skin effects at water depths greater than approximately 500 m further complicate the physicochemical behavior and lifetime modeling of gas bubbles [13]. Shipborn acoustic echosounding represents the state-of-the-art technique to remotely sense marine seepage. Until recently, singlebeam echosounders were operated to reliably sense gas seepage sites even beyond 2000 m water depth [14]. Today, wide coverage multibeam echosounder systems can be used for more efficient *in situ* and shipborn bubble detection [15,16,17] and even allow for bubble rise velocimetry adapted from visual computer vision [18]. However, the acoustic absolute quantification of seepage flux remains a very challenging task, and without the knowledge of bubble size distributions and rise velocity, the inversion of single frequency echosounder data into gas flux estimates remains ambiguous [19]. Moreover, acoustic analyses of individual bubble shape, small-scale bubble trajectory, rising speed, surface characterization, exact bubble size determination, and upwelling phenomena of ambient seawater are limited due to wavelength and resolution. This shortcoming can be overcome by high resolution *in situ* visual sensors that allow for measuring the bubble size distribution of a stream and the rise velocity of bubbles (see e.g., [20,21,22]). These numbers can then again be used to enhance acoustic inversion and bubble lifetime modeling and support regional acoustic inversions. In this contribution, we present first investigations towards a novel 3D sensor called bubble box to quantitatively investigate natural gas seepage bubbles in the oceans. It can be deployed on or towed across the seafloor using a TV-guided frame or an ROV (remotely operated vehicle) and is depth rated up to 6000 m. The goal of this work is automated determination of bubble size distribution and rise velocity as well as overall gas flux. The bubble box in its current design (see Figure 2 for sample images) allows for flux validation by capturing a reference volume at the top. It is equipped with a wide baseline stereo camera system and background illumination to capture the 3D shape of rising bubbles in order to obtain bubble characteristics for a certain vent, or ultimately, the statistics of vents in an entire area.

### State of the Art in Visual Characterization of Bubble Streams

Mechanical installations such as inverted funnels can help estimating an average flux but cannot provide the rise speed or bubble size distribution required by diffusion and dissolution models as discussed above. This information is also an input parameter of inverse acoustical methods to compute flux from large scale sonar surveys. On the other hand, in visual data it is possible to distinguish and track bubbles for rise speed and size distribution analyses, so that we discuss the state of the art in visual characterization of bubble streams in the next paragraphs.

**Figure 3:**
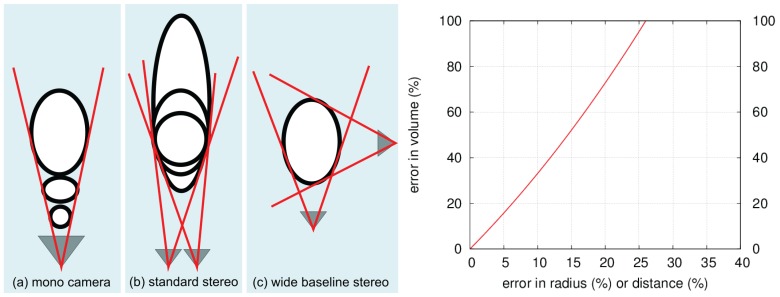
Different settings to observe bubbles: (**a**) For a monocular camera a large distant bubble looks the same as a small bubble closeby; (**b**) A small baseline stereo camera system can measure the distance of spherical bubbles, but the extent of ellipsoidal bubbles in viewing direction is very uncertain; (**c**) A wide baseline stereo system can determine the ellipsoidal shape well.

Several visual systems have been presented for *in situ* measurement of bubbles in the ocean. The systems differ in whether they are intended for shallow water and waves [20], for moderate depth [12,23] or for the deep sea [21]. Besides dedicated sensors, bubbles have also been analyzed from ROV cameras [24]. These systems have been primarily designed for capturing good data and require a lot of interactive manual work to process the data afterwards. All systems referenced above rely on a monocular camera. Monocular cameras are well suited for observing spherical bubbles, when the distance of the bubble to the camera is exactly known. However, even if the distance to the seepage source is measured carefully, the camera distance to individual bubbles in a stream may vary, as bubbles can show zig-zag motion or oscillation (*cf*. to [3]) and several bubbles can appear next to each other. By the intercept theorem from elementary geometry, it can be understood that an error of 25% in assumed distance results also in an error of 25% in estimated sphere radius and a volume error of almost 100% (*cf*. Figure 3). In order to remedy this problem, recently Wang and Socolofsky [22] have presented a stereo-camera system. The use of two cameras allows for measuring the distance of each bubble to the camera more precisely, even if it is not exactly in the center of an assumed corridor. Then, for spherical bubbles, the 3D sphere size can be computed from the observed circle in the image. As we will outline in the next section, the stereo configuration can however be improved for analysis of ellipsoidal bubbles (see Figure 1 for different shapes).

The problem is also related to image processing based on particle tracking in lab environments. Here, [25] compute the rise speed of the center of mass of bubbles in an aquarium using a mirror but do not perform volume or sizes estimates. Bian *et al*. [26] estimate a restricted ellipsoid model for a single bubble. Both studies do not report on how they handle the refraction at the air-glass-water interfaces, which has to be considered properly in multi-media photogrammetry (*cf*. e.g., to [27]). Towards this end, recently, novel techniques for modeling and calibration of distance, thickness, and normal of the interface of the camera housing have been proposed [28,29]. We carefully analyze the calibration issues for the proposed bubble box sensor. In terms of pure 2D image processing, [30] presents automated detection and shape estimation as well as tracking using a monocular camera. In the next section, we will present the details of our system and outline the design considerations.

## 2. Bubble Box Design and Calibration

The visual sensor that we entitle bubble box can be seen in Figure 2. The goal of the sensor is to measure rise speed, flux, shape, and bubble size distribution at the source position of methane or carbon dioxide seeps at the ocean floor. For this, the system must be both mobile and robust, such that it can be deployed by a remotely operated vehicle (ROV) from a research vessel. The body therefore consists of a steel frame of a 30 cm by 30 cm footprint on the ground (plus surrounding plate for carrying batteries), with approximately 1 m height.

### 2.1. Design Considerations

**Figure 4:**
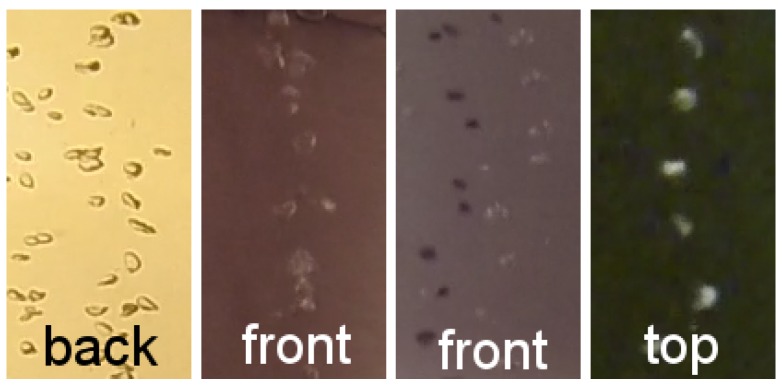
Different lighting configurations. By far the best results are achieved by back lighting, *i.e.*, using a diffuse light source behind the bubbles. This causes the bubbles to appear as dark contours. When using a diffuse light source from the same direction as the camera, the bubbles are less well distinguishable from the background. Using non-diffuse front lighting causes the bubbles to have shadows, which complicates the detection process. Another interesting option can be top lighting, which can be seen in the right image.

The box provides a vertical corridor where a bubble stream can rise through. To avoid horizontal drift of bubbles due to currents, the corridor is protected to the sides by acrylic glass. The corridor width of 20 cm was chosen to minimize boundary effects for thin bubble streams with a distinct source, when the box is centered on the source. Two of the four outer vertical faces of the corridor are made of white acrylic glass with strong scattering and little attenuation properties (6 mm polymethylmethacrylate, “Perspex”). These are illuminated from the back and function as bright, white background plates when the box is in operation (different illumination setting tests, e.g., from the front, from the top and from the back can be seen in Figure 4, and compare also to [21]). The two other vertical faces use transparent acrylic glass and contain a camera each. The system has been designed to carry robust deep sea cameras (1024 × 1024 pixel resolution machine vision cameras with 70${}^{\circ }$ field of view, controlled by a mini computer, in titanium housings with 10 cm diameter dome ports). Both cameras are triggered using a separate micro controller and send the images via a gigabit Ethernet connection to a small computer inside the pressure housing. Images are finally stored on a shock resistant 1 TB solid state disk. The system can be powered by the ROV that deploys it or as a standalone system and requires approximately 60 W (including lighting) during operation and negligible power during standby. Storage and power are sufficient for single day missions of many deployments on several seeps but are currently a limiting factor for long term monitoring. We have also built a light-weight version using (1280 × 720 pixel resolution) GoPro Hero3 cameras in Polyoxymethylene housings for shallow water). The cameras are arranged in a way that they observe the bubble corridor from a distance of 10 cm and from two approximately orthogonal perspectives. This way, each dimension of a bubble is observed by at least one of the cameras. In contrast, in standard stereo settings, where both cameras are next to each other and share almost the same perspective, noisy observations of bubble rims lead to high uncertainty of the bubble extent in viewing direction (see also Figure 3).

The target capture rate of the system is 120 Hz. The observation corridor is approximately 20 cm high, which means that a bubble rising with 25 cm/s (compare [9] for a thorough discussion on rise speeds) in the center of the corridor will be photographed approximately 100 times. Faster bubbles will be photographed slightly less frequently but can still be evaluated. The exposure time has to be selected such that a bubble moves less than half a pixel. In the scenario above, this is approximately 0.1 mm and leads to an exposure time of less than 5 ms. We are generally interested in observing bubbles larger than 0.5 mm in diameter (that have enough buoyancy to detach from the sediment and rise towards the surface). The minimum bubble size both camera systems can observe is therefore in the order of 0.5 mm in diameter. If smaller bubbles need to be observed (e.g., created from exploding bigger bubbles), different lenses or cameras with higher resolutions could be used, but this is out of scope of this article.

The acrylic glass walls are each illuminated by a high-powered LED from the back, which is synchronized to output all energy during the exposure time of the cameras. To make the system more lightweight/mobile, it is also possible to illuminate from the back using e.g., ROV lights or diver-provided illumination.

### 2.2. Calibration

In this contribution, the term camera calibration stands for geometric calibration, *i.e.*, determining which pixel corresponds to which 3D ray in water. In the case of a stereo camera rig, where the cameras are rigidly mounted, this means that the cameras need to be synchronized in order to gain corresponding images of the bubble stream at the same time. This, in turn, allows for determining the extrinsic parameters, *i.e.*, the position and orientation of one camera with respect to the other. Additionally, intrinsic parameters like focal length, but also of the underwater housing need to be calibrated.

#### 2.2.1. Synchronization

The deep sea camera system is hardware triggered and will capture images at the same time. In order to temporally calibrate the lightweight stereo system of GoPros, and later to match bubbles across a stereo pair, the two video streams need to be synchronized with an accuracy down to a single frame even at frame rates of 120 frames per second. The GoPro Hero 3 black edition does not allow synchronized capture with that accuracy. To allow temporal alignment of the videos in postprocessing, a short blinking light (flash) is presented such that both cameras can see it. Then, the streams are synchronized manually by importing both streams into the open source video editing software cinelerra (http://cinelerra.org) and aligning the light on/off frames. The corresponding left and right images were put together into a single (synchronized) stereo image that was then later used for further processing for calibration and bubble measurement. This method allows to synchronize the streams but requires some manual work.

#### 2.2.2. Stereo Calibration

The perspective camera model in air describes how a camera projects 3D points into the image plane, after intersecting the center of projection (single-view-point camera). In this work, a standard model with focal length, principal point, and radial distortion [31] is used and calibration is performed according to the method described in [32]. It relies on checkerboard images that are captured from different points of view, including different distances from the camera and different angles relative to the camera’s optical axis.

**Figure 5:**
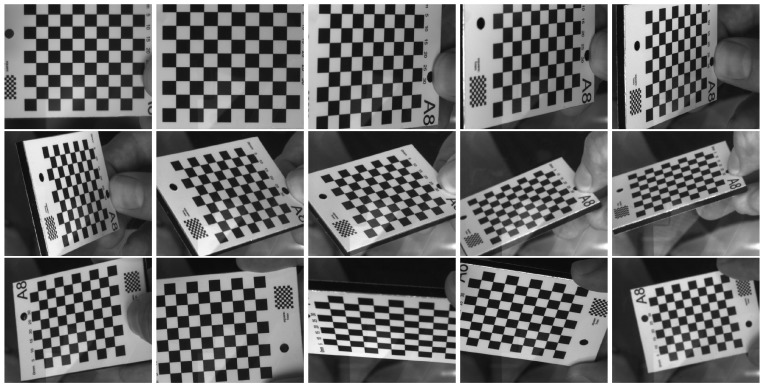
Calibration in water, sample input images from different points of view.

When using a camera underwater, it needs to be enclosed in an underwater housing viewing the scene through a glass port. In case of flat ports, the different media (water, glass, air) cause the light to be refracted, and hence the perspective camera model to become invalid, introducing a systematic measurement error. The magnitude of this systematic measurement error depends on the camera-glass configuration and the distance of objects. Dome ports, semi-spherical glass housings, do not suffer from refraction, when the camera center is aligned with the sphere center. On the other hand, cameras behind flat ports that are centimeters away from the glass, possibly even with an inclination angle between camera and glass, produce a large measurement error if refraction is ignored. Each such underwater camera system that is to be used for measurements needs to be carefully evaluated to see if refraction needs to be modeled explicitly (refer for example to [28,33,34] for calibration methods). Treibitz *et al*. [33] showed that the caustic size is a measure for deviation from the single view point camera model. In particular, for the center bubble area in a low resolution GoPro mounted very close to the interface, the caustic is negligible and perspective calibration can be used. This however depends on the actual camera and housing used as well as on the setup and the introduced error has to be analyzed carefully. In any case, the cameras need to be calibrated by capturing a set of underwater checkerboard images as in Figure 5. Then, depending on the expected caustic size, either the perspective calibration method is used (not explicitly modeling refraction [35]) or a refractive method, e.g., [28] can be used to determine housing parameters like glass distance and inclination.

Afterwards, the relative translation and rotation between the two cameras need to be determined by using checkerboard images that are completely visible in both cameras.

## 3. Bubble Stream Characterization

**Figure 6:**
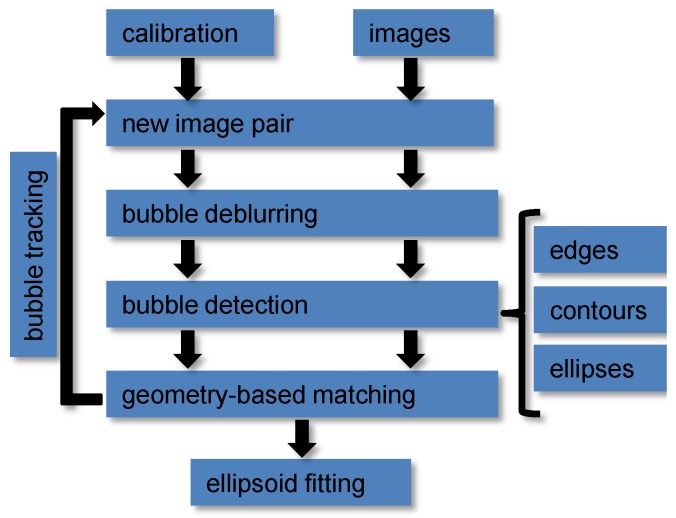
Flow diagram of the stereo bubble box data processing steps.

The previous section described the hardware and the calibration routines for the bubble box. Once a set of bubble images has been captured, automated methods for image processing are required that estimate properties like overall volume, rise velocity, and size distribution. In a preprocessing step, the images’ regions of interest (ROI) are determined and in an optional step, possible blur is removed. Then, the bubbles are detected and matched across the stereo image pairs. This allows for computing ellipsoids for each bubble. Finally, it is of interest to track bubble streams over time to determine 3D bubble paths and to compute rise velocities. Refer also to Figure 6 for an overview of the method.

### 3.1. Preprocessing—Automatic Region of Interest Selection

**Figure 7:**
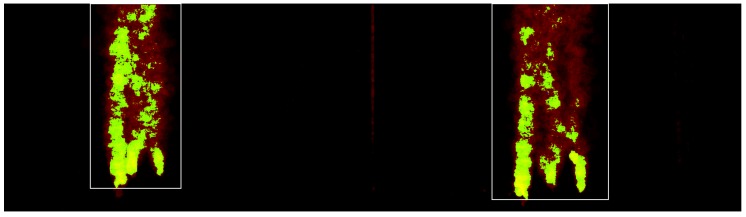
Heat map showing the automatically determined bounding boxes (**white**) for bubble detection in the left and right stereo images.

It is assumed that only the bubbles move within the images and that they occupy a fairly constant region of interest (ROI). However, bubble detection is based on gradients within the images and will also fire on other possible structures like, for example, markers for measurement as can be seen on the diffuse acrylic plates in Figure 2. Therefore, it is important to have a region of interest in both images that contain the bubble plume only. This can either be determined manually or by utilizing the assumption that the only moving objects in the images are the bubbles. This movement can then be detected using optical flow methods [36]. The areas of the images, where movement is measured over time are then accumulated in a heat map and the region of interest showing the bubbles to be measured is the resulting surrounding rectangle (refer to Figure 7). For the evaluation presented in this article, a static ROI has been computed for each sequence and was not changed during the sequence. In addition to determining the ROIs, computing the heat map by using optical flow methods allows to initially predict bubble motion between consecutive frames, which will be used for tracking bubbles across time. However, the optical flow estimation results contain outliers due to sporadic large bubble displacements, such that the results usually cannot be utilized directly for bubble tracking over time.

### 3.2. Preprocessing—Bubble Deblurring

As already stated in the introduction, the frame rates for capturing gas bubbles need to be high and exposure times need to be short in order to avoid motion blur in the images. Consequently, the capture of bubble streams requires strong illumination. For long term observations with limited energy at the sea floor, or for lightweight deployment of the system without integrated lighting and battery (*i.e.*, mobile lights from divers), too little light might be available for good quality images. To some extent, this can be compensated by longer exposure times that lead to moderate motion blur or a larger aperture that leads to a smaller depth of field [37]. However, larger apertures can cause the bubble to be outside the depth of field and will hence cause defocus blur.

Both blur effects skew the measurements of the bubble’s shape and size, which are important measures in bubble box applications. The fidelity of images acquired under less-than-perfect conditions can be improved by blind deconvolution techniques with a gradient sparsity prior. This will compensate for the influence of motion and defocus blur to some extent. This section focuses on the practical application of the algorithm, for more details, see results already published in [38].

Blind deconvolution is based on the following image model. The observed image *O* is formed from the undisturbed image *S*, convolved with the blur kernel or point spread function (PSF) *B* and additional noise *n*:

(1) O=S⊗B+n

Blind deconvolution means that both *S* and *B* have to be recovered from a single measurement *O*. This problem of recovering these two unknowns (*S*, *B*) from one measurement (*O*) is ill-posed in general as shown in [39]. However, by using additional constraints or exploiting special properties, solutions can be found [39,40]. For instance, [41] uses a heavy tailed gradient sparsity prior, designed for the restoration of natural images [39]. Bubble box images typically show a uniform background with low gradient values and bubbles with a stronger gradient rim. Consequently, also for bubble sharpening, a MAP (Maximum *a posteriori*)-estimation gradient sparsity blind deconvolution algorithm (*cf*. to [41]) is suitable. The formulation is based on the Bayesian framework and uses the Maximum *a posteriori* (MAP) principle as its foundation. For the MAP principle, the latent distribution of the observed image *O* is denoted as P(O). P(S) and P(B) denote the *a priori* distributions of the undisturbed image and of the blur kernel, both with applied priors. The algorithm recovers the undisturbed image *S* and the blur kernel *B*, by finding their MAP, dependent on *O*:

(2) $$\begin{array}{c} \begin{array}{cc} P(S,B|O) & =\frac{P(O|S,B)P(S,B)}{P(O)}\\ & \propto P(O|S,B)P(S,B)=P(O|S,B)P(S)P(B) \end{array} \end{array}$$

while P(O|S,B) follows a Gaussian distribution with parameter *γ* that encapsulates the Gaussian’s standard deviation:

(3) $$P\left(O|\left(S,B\right)\right)\propto e^{-\frac{\gamma }{2}{∥S⊗B-O∥}^{2}}$$

On the recovered signal *S* and the blur kernel *B*, the regularizers Q(S) and R(B) are employed. *Q* controls the gradient sparsity prior on the image and *R* the assumptions on the blur kernel. This allows the recovery of the blur kernel and sharp image from the blurred image. For a more detailed description of the algorithm, see [41].

### 3.3. Bubble Detection

**Figure 8:**
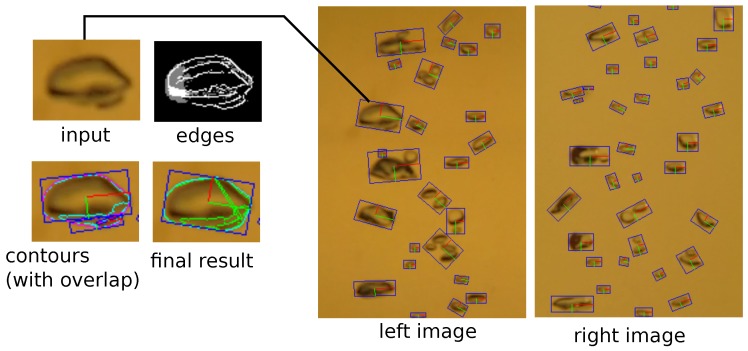
Detected bubbles in image ROIs. In **cyan**: contour around the bubble rim. In **blue**: rectangles describing the ellipse.

Within the region of interest in an image, the bubbles are to be detected and tracked automatically (refer to [30] for a more detailed description of the bubble detector and tracker). For this, the images are interpreted as a function mapping a gray value to each pixel. In this function, gradients can be computed and used for line-detection with the Canny edge-detector as in [21]. The edges are then used to determine the bubble contours by tracing the convex hull of the connected components. Extra contours inside the bubbles are rejected, by checking for overlapping detections. The final ellipses are fit into the remaining contours using a method by [42]. Figure 8 shows intermediate and final results of bubble detection.

At this point, the image ellipses around the detected bubbles would allow the calculation of the volume by assuming that each bubble is a rotationally symmetric, 3D ellipsoid. This method based on a monocular camera only is often used in the literature for bubble volume estimation. An additional assumption required in this case is that the distance between camera and bubble is constant and known, thus allowing to convert the measured volume to metric units, *i.e.,* computing a factor, which determines how many pixels correspond to one millimeter. This method works well if the bubble box contains only one bubble plume at a precisely known distance to the camera. However, deviations from the assumed distance affect the estimated volume with an error in the third power of the error of the distance, resp. conversion factor (see Figure 3).

### 3.4. Stereo Matching and Ellipsoid Triangulation

In the case of more than one bubble plume being measured in the bubble box or in case the distance between bubbles and camera is not known with high accuracy, a wide-baseline stereo camera rig is used to accurately triangulate 3D bubble positions and reconstruct their ellipsoidal shape approximation. This is achieved by utilizing the knowledge about the calibrated rig to compute 3D cones of sampled rays for each bubble rim, which can then be intersected. Consequently, the exact distance of each bubble from the two cameras is computed. This allows not only precise volume measurements, but also to reconstruct the 3D information of single bubbles in the plume and track its 3D movement upwards.

**Figure 9:**
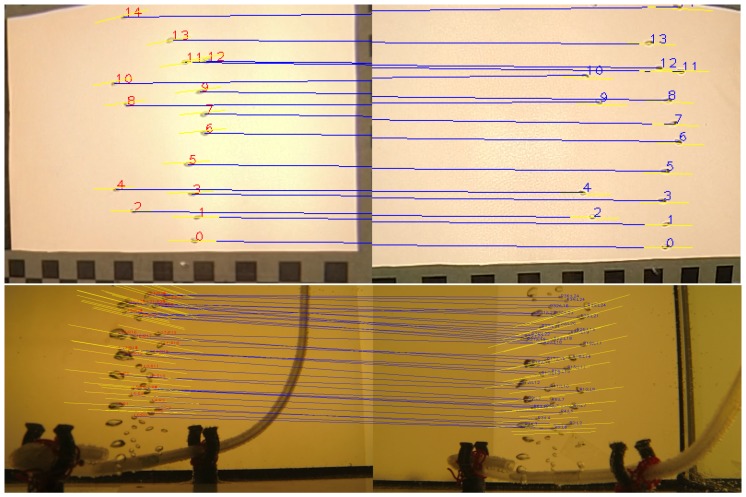
Exemplary results for stereo matching. **Top**: few bubbles, matching works very well. **Bottom**: many bubbles with overlap, where the matching procedure will make some errors.

The method then works as shown in Figure 6. The stereo rig calibration and a series of input images serve as input. In each image pair, the bubbles are detected with the method described in Section 3.3. Due to the wide baseline, it is not possible to match the bubbles according to their shape (refer to Figure 8), so matching has to rely on two-view geometric constraints. For perspective cameras, this can be done using epipolar geometry [43], based on the idea that a point in one image has to lie on a corresponding line in the second image. However, in presence of refraction, epipolar lines become curves. In this case, we use piecewise, linear approximations of the epipolar curve. Note that for this kind of geometric matching, the rig calibration needs to be very accurate, otherwise, the computed epipolar lines will not intersect the matching bubbles. The distances to the epipolar lines are used as weights. Then, the two sets of bubbles form the two sets of a weighted, bipartite matching problem (see Figure 9 for results).

**Figure 10:**
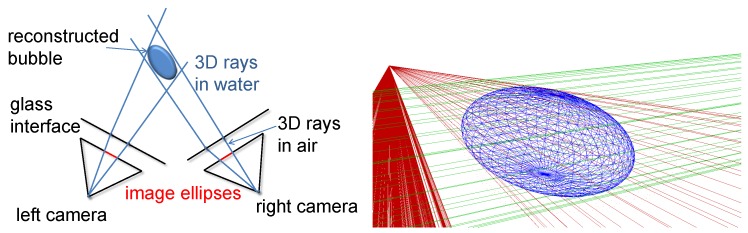
**Left**: ellipsoids are computed by determining the boundary rays corresponding to the contours in the images. **Right**: when viewed in 3D, so-called boundary cones are defined when discretely sampling the points on the contours in the images. The red cone is from the first camera and the green cone from the second camera. The blue ellipsoid is fitted to lie inside the cone intersection. Note that the computation of 3D rays does not require the projection of 3D points into the images, which is infeasible when using the refractive camera model.

With the stereo correspondences, the 3D position and ellipsoidal shape of the bubbles are triangulated, by utilizing the calibration information about the camera configuration. For the purpose of shape reconstruction, different approaches are feasible. [26] uses an analytic approach of calculating the parameters, which delivers exact results and also requires exact synchronization. Note that shape-from-silhouette approaches [44] are also an interesting alternative in general; however, they usually require a large number of projections of 3D points into the images. When using a refractive camera model, such approaches are prohibitively expensive. Therefore, the described system intersects the bubbles’ viewing cones: for each bubble correspondence in a stereo image pair, the outer contour is sampled discretely and for each sample, the corresponding ray in space is computed. The set of 3D rays for each bubble contour forms a characteristic cone (see Figure 10). The bubble is then reconstructed by fitting an ellipsoid on the inside of the cone intersection. Note that in the presence of noisy observations, this kind of cone intersection is more ambiguous in small baseline stereo camera setups than in our wide-baseline setting (compare to Figure 3).

Compared to estimating the bubble volume by assuming certain fixed distance for each bubble from the camera, the approach described in this section triangulates the 3D bubble position and determines its 3D ellipsoidal shape. From the ellipsoid, the bubble’s volume can be computed directly. In case of failed matching, the bubble volume can still be approximated from one of the stereo images by assuming a rotationally symmetrical bubble at an average distance (traditional monocular assumption).

### 3.5. Bubble Tracking

To reconstruct the motion of the bubbles, they are tracked over the image sequence, *i.e.*, over time. Due to the bubble usually following a constant upwards motion with certain deviations to the left and right, it is possible to specify an upwards and a sideways motion constraint, thus allowing the determination of a set of possible matching candidates between two consecutive images. The matching candidates are assigned a weighting factor based on the motion constraints, which leads to a minimum-weighted, bipartite matching problem, which is a classical graph problem and can be solved by the Hungarian algorithm [45]. When combining the correspondences over time, the entire trajectory of the individual bubbles can be inferred.

## 4. Assessment

Before evaluating the performance of the presented system, results of the optional deblurring step will be shown. Then, the described method will be evaluated on synthetic and real data.

### 4.1. Deblurring

**Figure 11:**
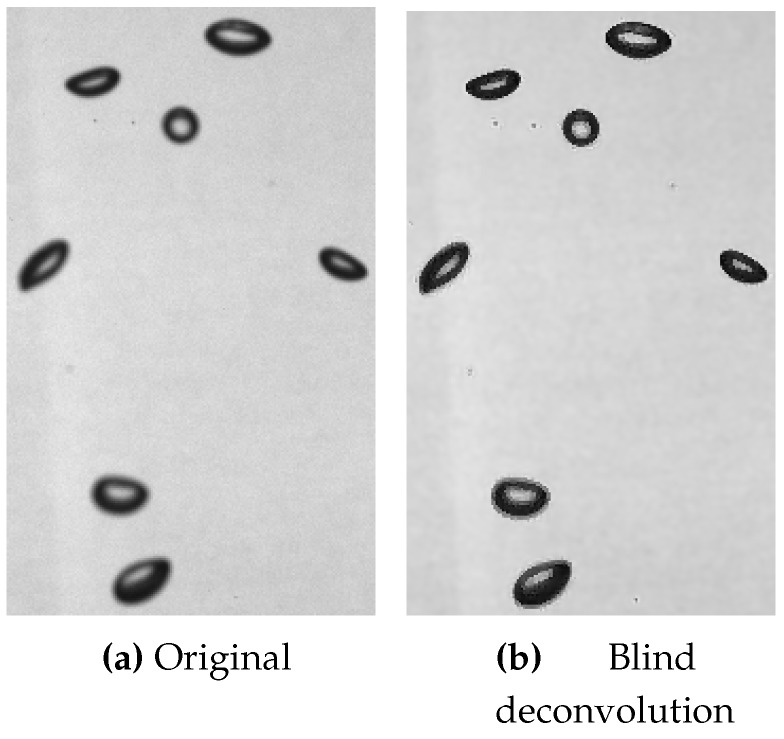
Experimental results of blind deconvolution on an input image with defocus: (**a**) shows the input image and (**b**) the result of gradient sparsity blind deconvolution.

Figure 11 shows results of the gradient sparsity algorithm as suggested in [41], which produces good results on the input image with only slight defocus (Figure 11a).

**Figure 12:**
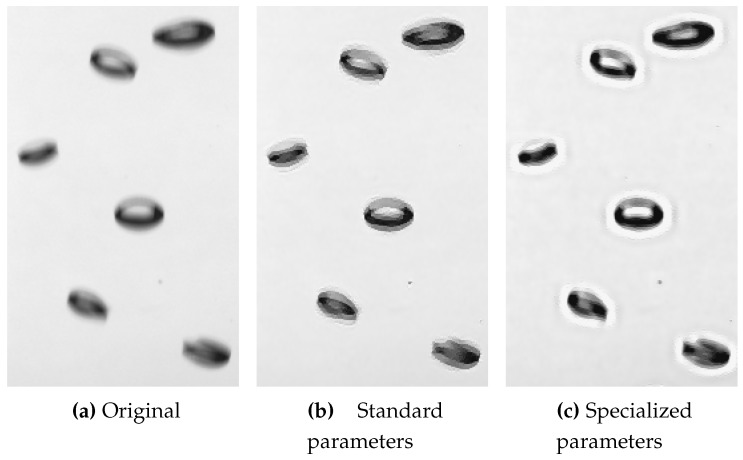
Cropped results of blind deconvolution on an input image with motion blur: (**a**) shows the cropped original image; (**b**) gradient sparsity blind deconvolution with standard; (**c**) gradient sparsity blind deconvolution, with a specialized parametrization for bubble box images, see Section 4.1 for details.

For motion-blurred images, the deblurring of [41] also shows a good improvement in sharpness, but remains incomplete, see Figure 12b. Clearer contours can be achieved as described in [38], *i.e.*, by using 2.5 times the weight for the sparsity prior of the blur kernel (compare Figure 12b,c). In addition, notice the strong halo artifacts around the bubbles caused by this parameter setting. However, they do not disturb the bubble detection, due to the gradient on the bubble rim being increased, while the gradient between background and halo is low in comparison. Therefore, to compensate motion blur, these specialized parameters should be employed for bubble images.

**Figure 13:**
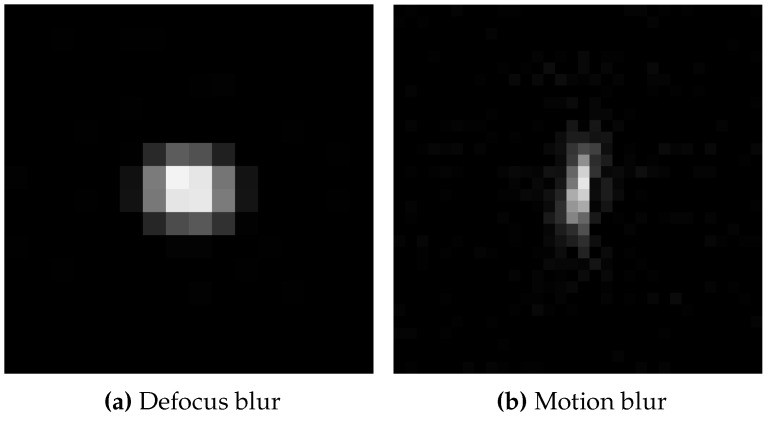
Blur kernels recovered by blind deconvolution, (**a**) shows the 15×15 blur kernel recovered from the defocused image in Figure 11a, while restoring Figure 11b; (**b**) shows the 32×32 blur kernel recovered from image with motion blur in Figure 12a, while restoring Figure 12b. Values between 0 (**black**) 1 (**white**), values scaled for better visibility.

The correct restoration is confirmed by the recovered PSFs in Figure 13 with its characteristic shapes for defocus (cf. to Figure 13a) and upwards motion blur (cf. to Figure 13b)

The results of deconvolution indicate that the fattening of bubble contours caused by motion and defocus blur, which leads to an overestimation of the bubble size, can be reduced. This has also been confirmed in a detailed analysis in [38]. Blind deconvolution is computationally expensive and the run-time for the gradient sparsity algorithm in a Matlab implementation is up to 10s on an image with 261 pixels width and 612 pixels height. A hybrid approach that uses blind deconvolution to estimate the PSF once and uses a non-blind deconvolution with a lower run-time for the following images, is a feasible and practical approach.

### 4.2. Bubble Simulator

In order to verify the developed algorithms on ground truth data, a bubble simulator was used that rendered synthetic images with known ground truth. For this, a simplified model for the bubbles is implemented. Randomized 3D ellipsoids are generated that move upwards on a randomized path. Additionally, the length of the ellipsoids’ main axes change from frame to frame without changing the volume. Thus, for each frame to be rendered, a known 3D representation with ellipsoids is known in addition to the exact 3D path a bubble took.

For rendering the images, we aim to simulate the back-lighting of the bubbles as shown in Figure 4. Bubbles do not follow the standard computer graphics Blinn–Phong shading model [46], but instead feature a darker rim and a bright core (see Figure 1). The reason for this darker rim is that while light reaching a bubble surface is refracted according to Snell’s law, if the angle of incidence is small, total internal reflection occurs. Our approximation of this process is to model the color of the bubbles with the angle of incidence of the cast viewing ray. This allows rendering reasonable test images using a simple raycaster, without requiring the complexities of a full, non-sequential raytracer.

For comparison, the bubble stream is rendered from three points of view. The first two views are set in the wide baseline scenario with a $90^{\circ }$ angle, while the first and the third camera feature the small-baseline scenario with a $20^{\circ }$ angle. All three cameras view the same set of bubbles, and hence the simulator allows for the comparison of performances of the proposed wide-baseline scenario, the small-baseline scenario, and the use of a monocular camera. Each camera observes the bubbles using the refractive camera model and during bubble size computation, refraction at the underwater housing is modeled explicitly.

**Figure 14:**
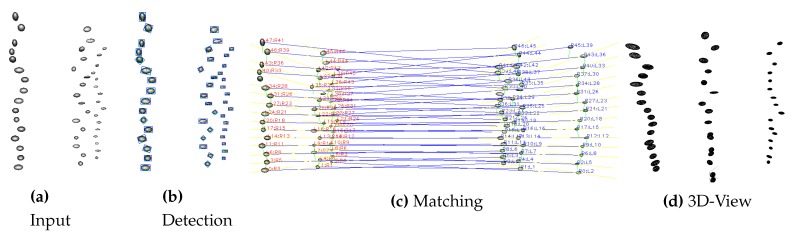
Exemplary simulator images. From **left** to **right**: input image, bubble detection result, matching result, and 3D view of the original 3D bubbles. In case of the matching image (**c**), the blue line indicates matching bubbles and the yellow lines indicate the local approximations of the epipolar lines.

Using the simulator, we generated a sequence of 100 images with three bubble plumes. For each bubble, the ground truth position, volume, and size are known, therefore, the performance of the proposed system can be evaluated on this data. Figure 14 shows an exemplary input image of the simulator on the left, followed by a bubble detection result, a matching result, and a 3D view of the bubble plumes.

**Figure 15:**
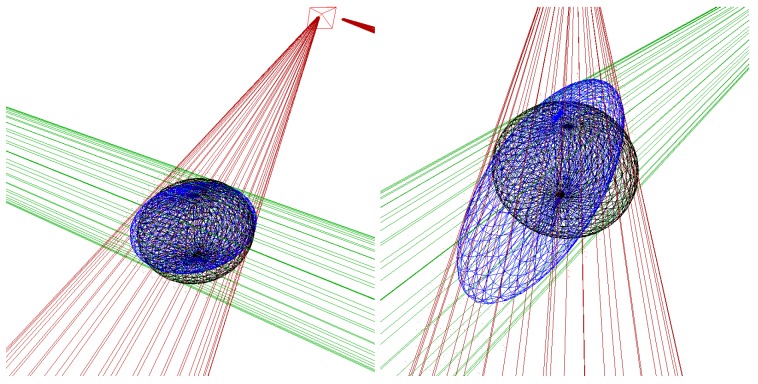
Ellipsoid triangulation from wide and small baseline. **Left**: wide baseline scenario, where blue shows the computed ellipsoid and black the ground truth ellipsoid. The green and red lines show the 3D cone limiting the ellipsoid. **Right**: small baseline scenario. Note how the blue ellipsoid cannot be determined uniquely due to the elongated intersection of the red and green viewing cone.

**Figure 16:**
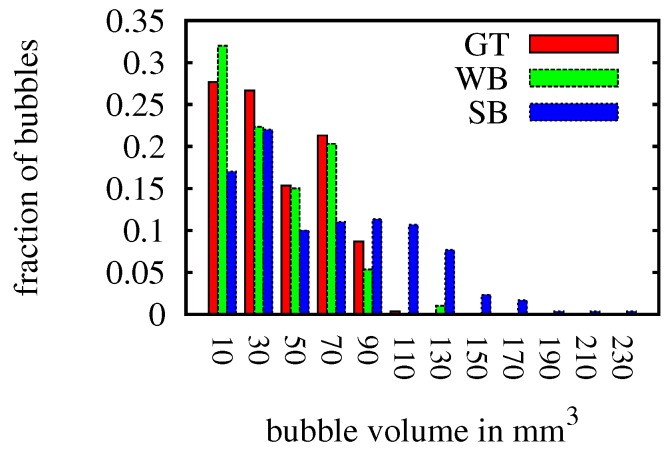
Histogram showing the bubble volume distribution on synthetic data computed in the wide-baseline scenario and the small-baseline scenario compared to ground truth.

Table 1 shows a summary of the results over all images for the wide-baseline scenario (WB), the small-baseline scenario (SB), and using the monocular camera with an assumed pixel-to-mm conversion ratio (Mono). The ground truth volume of the bubble stream is higher than the estimate for the wide-baseline scenario by our algorithm due to some mismatches and therefore missed bubbles. Note that the average bubble volume was estimated accurately, showing that the ellipsoid computation at the $90^{\circ }$ configuration yields good results. The velocity of the generated bubbles was higher than in real bubble scenarios, but was also estimated accurately. The results of the small-baseline scenario demonstrate the major problem of this method. Matching accuracy was comparable in both scenarios, but when computing the ellipsoid, bubble size is usually over-estimated due to ambiguities in bubble shape. This is demonstrated on an exemplary bubble in Figure 15, where the left image shows the ellipsoid in the wide-baseline scenario and the right image shows the ellipsoid computed in the small-baseline scenario (compare also to Figure 3). The histogram of bubble volumes in Figure 16 confirms this observation. While the results of the wide-baseline scenario match the ground truth histogram well, in the small-baseline setting the bubble volume in general is overestimated in our experiment. Table 1 also gives some results using a monocular camera. In this case, a fixed pixel-to-mm conversion ratio is assumed, to estimate bubble volume with the assumption that all bubbles have the same distance to the camera and are rotationally symmetric.

**Table 1:** Results on synthetic data. The first column shows the ground truth results. WB stands for wide-baseline scenario, SB for small-baseline scenario and Mono shows an estimate for using one camera only based on a pixel-to-mm conversion ratio.

	Ground Truth	WB	SB	Mono
**Synthetic Data**				
volume	127,85.72 mm${}^{3}$	11,391.93 mm${}^{3}$	18,823.21 mm${}^{3}$	15,028.2 mm${}^{3}$
average volume	42.62 mm${}^{3}$	39.83 mm${}^{3}$	66.04 mm${}^{3}$	52.18 mm${}^{3}$
average velocity	35.94 cm·s${}^{-1}$	36.00 cm·s${}^{-1}$	36.16 cm·s${}^{-1}$	-
# bubbles	300	286	285	288

### 4.3. Test Setup with Air Bubbles in Water

**Figure 17:**
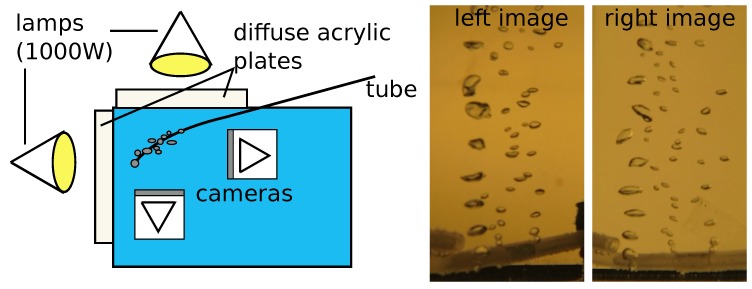
**Left**: test setup for evaluation of the system. Two back-illuminated planes provide a bright background behind an air bubble stream generated by a small tube. The cameras are arranged in a wide baseline setting viewing the scene from $90^{\circ }$ different perspectives. **Middle** and **right**: exemplary images showing the produced bubble streams with small, ellipsoidal bubbles and large bubbles with arbitrary shapes.

The camera setup of the bubble box has been tested prior to building the actual box using lab experiments in a fish tank filled with water (refer to Figure 17). Inside of the water, three bubble streams were produced. Note that in order to test the automated data processing algorithms, a simple stream of air coming out of a flexible tube is sufficient for which the actual overall volume was measured using a measuring cup. In order to illuminate the bubble streams, two adjoining glass walls of the fish tank were equipped with white acrylic glass that strongly diffuses the light. Both acrylic planes were lit with 1000 W halogen lamps. Inside the fish tank, two GoPro 3 cameras were set at a $90^{\circ }$ angle at a distance of about 25 cm to the bubble streams. The GoPro cameras have a very small distance between glass and camera and the cutouts used in this example only utilize a small opening angle of the overall image. For this cutout, the caustic and therefore the deviation from the single-view-point camera model is very small and the cameras were calibrated perspectively using checkerboard images captured underwater.

By manipulating the tube, it is possible to create many or few, small or large bubbles (refer to Figure 17). However, for our real-world applications, we are mainly interested in bubble sizes of several millimeters, which maintain approximately ellipsoidal shapes.

The system was evaluated on a sequence of 600 stereo images and the summarized results can be seen in Table 2. The proposed method underestimates the flux, which can be explained by bubbles that could not be detected properly and are therefore very difficult to match with the geometric matching procedure and need to be discarded. In the last column, we therefore present an approximate estimation of the flux of the missed bubbles. This flux was estimated using the monocular approach, *i.e.,* by assuming a constant distance between camera and bubbles and a known pixel-to-mm conversion ratio. Note, however, that, in this case, the distance between camera and bubbles is unknown and differs between the bubbles inside a plume but also between the three different outlets, and we therefore can only roughly estimate the volume. Additionally, bubbles that were not detected individually, but were overlapping are not compensated for.

**Figure 18:**
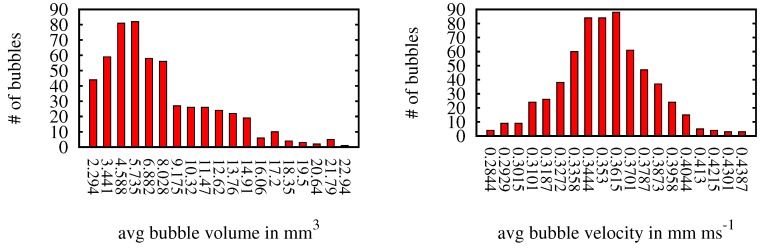
Histogram showing volume and velocity distribution of the bubbles.

**Figure 19:**
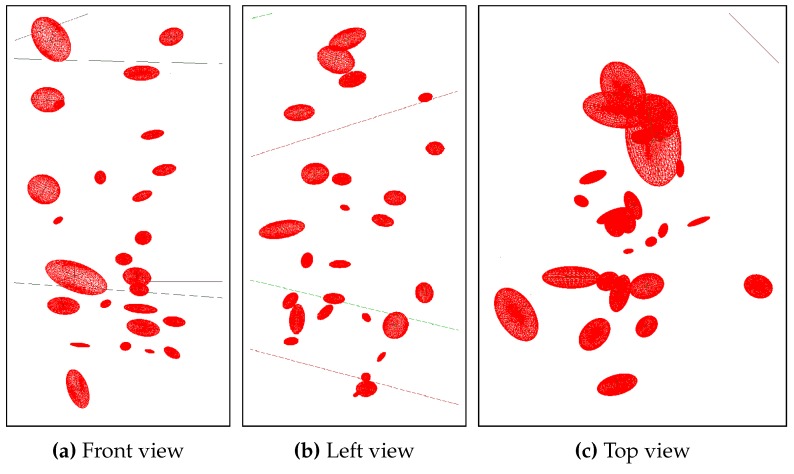
3D-Reconstruction of a bubble stream.

Figure 18 shows histograms of the bubble volume distribution and the bubble velocity. Throughout the sequence, there were three bubble streams one of which consisted of larger bubbles, while the other two contained smaller bubbles. This is reflected in the one-sided volume histogram. The velocity is normal distributed for all bubbles and Figure 19 shows three different views of the reconstructed 3D bubbles.

**Table 2:** Results on real data image sequence.

	Measured	Results	Comment
**Real Data**			
flux	4.177 mL·s${}^{-1}$	2.504 mL·s${}^{-1}$	estimated volume of missed bubbles 0.291–0.7488 mL·s${}^{-1}$
velocity	-	36.135 cm·s${}^{-1}$	-

## 5. Discussion

The first results presented in the previous section show that the system has the potential for fully automated computation of bubble size distribution, volume, and rise velocity. In case of non-ideal lighting conditions, the images can be improved by the deconvolution method to avoid biased size estimates.

When considering the bubble stream characterization results, it can be seen that the proposed method works well on the synthetic image sequence, while it over-estimates bubble size when using the small-baseline camera setting on the same bubble stream.

**Figure 20:**
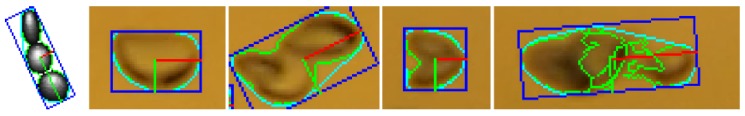
Exemplary bubble detection results. Green shows the detected contours, cyan, the convex hull, and the blue rectangle the final ellipse fitted around the detected contour. From **left** to **right**: erroneous detection on synthetic image; correct detection on real image; erroneous detection of two or three bubbles as one on real image; detection of two bubbles as one on real image; detection of two bubbles as one on real image with additional inner contours.

On real data however, the proposed method under-estimates the bubble flux, which is due to bubbles not being detected properly mainly due to overlap and stereo matching ambiguities. Overlapping and occluding bubbles in the images are a challenge for all bubble quantification methods, and some examples are shown in Figure 20. In the future, we plan to improve our image processing methods to further disambiguate those cases. For example, we plan to experiment with active contour methods for separating partially overlapping bubbles (refer to [30]). Additionally, the wide baseline camera setup can be utilized to detect bubbles that are completely occluding each other in one image. In those cases, both individual bubbles should be visible in the second image and our ellipsoid intersection method should be able to compute a fairly good estimate of both bubble volumes by intersecting the viewing cones. However, for this, the two bubbles in the second image need to be correctly matched to the overlapping bubble in the first image, *i.e.*, one-to-many and many-to-many matches need to be handled. Additionally, looking at longer bubble trajectories rather than a single stereo pair could allow to further disambiguate overlap or occlusion situations in the future. It should, however, be noted that, compared to a single camera or small baseline system, chances for resolving occlusion scenarios are much better as one of the cameras will see the occluded bubble.

Where previous 2D methods had to assume a certain symmetric ellipsoid model, it could be shown that it is possible to drop this requirement and rather exploit the two different perspectives onto the ellipsoid, in order to estimate shape and volume. Although the wide baseline stereo setting requires slightly more calibration and synchronization efforts, the approach of using a monocular camera has its own disadvantages: in practical applications, it is very difficult or even impossible to estimate the correct factor for converting pixels into millimeters because this factor depends on the distance between camera and bubbles (20cm×20cm corridor in the bubble box). However, errors in this factor are reflected with the third power in the resulting bubble volume (see Figure 3).

## 6. Conclusion

In conclusion, the proposed method still has some shortcomings in case of dense and multiple bubble plumes, where a lot of bubbles overlap in the images, but we suggest our measurement and automation will operate with high accuracy and reliability at single bubble seepage sites. Additionally, the hardware setting has the potential to disambiguate overlapping bubbles in the images. Future work will therefore focus on improving bubble detection and stereo matching. Also, the amount of available storage space is one of the limiting factors of the system. A long-term goal is therefore to do as much data processing as possible on site in order to eliminate the need of saving complete images.
